# Association between follicle size, endometrial thickness, and types of ovarian stimulation (*Clomiphene citrate* and Letrozole) with biochemical pregnancy rate in women undergone intrauterine insemination

**DOI:** 10.1186/s13104-023-06529-2

**Published:** 2023-10-24

**Authors:** Anita Rachmawati, Sofie Rifayani Krisnadi, Shasya Aniza Santoso, Annisa Dewi Nugrahani

**Affiliations:** Department of Obstetrics and Gynaecology, Faculty of Medicine, University of Padjadjaran – Dr. Hasan Sadikin General Hospital, Pasteur No. 38, Bandung, West Java 40161 Indonesia

**Keywords:** *Clomiphene citrate*, Endometrial thickness, Follicle size, Intrauterine insemination, Letrozole

## Abstract

**Objective:**

There was also a lack of data regarding the effect of follicle size, endometrial thickness, and ovarian stimulation as predictors of intrauterine insemination (IUI) success rate in Indonesia, especially in the Aster Clinic and *Bandung Fertility Centre*. This study was performed to explore the relationship between follicle size, endometrial thickness, and types of ovarian stimulation (Clomiphene citrate/CC vs Letrozole) with biochemical pregnancy rate in women undergone IUI. We performed a case–control study in 122 women aged 20–40 years with unexplained infertility who had completed the IUI program for a maximum of three cycles. Data were extracted from medical records. Independent T-test and multivariate analyses were used to analyse the difference between variables using IBM SPSS 24.0. P-value < 0.05 was considered statistically significant.

**Result:**

Follicle sizes of 18–22 mm in both Clomiphene citrate (CC) and Letrozole groups were shown to increase biochemical pregnancy rate (P = 0.001). There is no relationship between endometrial thickness and pregnancy rate. Biochemical pregnancy rate in women using Letrozole was 1.513 times higher than women using CC. The follicle size of 18–22 mm and using Letrozole rather than CC as ovarian stimulators are predictive factors associated with a higher pregnancy rate in women undergone IUI.

## Introduction

Infertility is estimated to affect 8–12% incidence among reproductive-age couples and shows an increasing trend. It is estimated that 1 in 7 couples in western countries experienced infertility, compared with 1 in 4 couples in developing countries [1–8]. Unexplained infertility (UI) occurs in 15% of all infertility cases. In women, UI is associated with advanced age, lower BMI, lower endometrial thickness, and poorer ovarian reservation testing [1–8]. In addition, infertility in women could rise to many psychological problems. Therefore, this condition is one of important health issues that must be addressed to prevent variety of adverse outcomes. [9, 10]

Regarding this issue, various techniques for treating infertility have been developed using assisted reproductive technology, such as intrauterine insemination (IUI) with controlled ovarian hyperstimulation (IUI/COH). The success rate of the IUI/COH method reported was about 11–16.4% [11–26]. Several factors that influence the success rates of IUI/COH, including: (1) type of stimulation, such as the use of Clomiphene citrate (CC) and Letrozole; (2) timing of ovulation after trigger shot was administered as determined by measurement of the dominant follicle (follicle size); and (3) endometrial thickness [20–26].

IUI is performed using a variety of ovarian stimulators such as CC and Letrozole. CC and Letrozole have become the drug of choice since CC is the first-line regimen, Letrozole is one of the first-line regimens for inducing ovulation in IU cases, and they are both inexpensive. According to previous studies, Letrozole was superior to CC [20–26]. The size of the follicle is also a factor considered to become as predictive factors of the IUI success rate. Multiple studies have demonstrated that the ideal follicle size to improve the likelihood of pregnancy is 18–22 mm [23–25]. In addition to the effect on follicle size, ovarian stimulation is suggested to also increase the endometrial thickness, which has a beneficial impact on the pregnancy rate. The optimal endometrial thickness to enhance the pregnancy rate in IUI is 8–10 mm, but the conclusion remains unclear in many literatures. This makes the choice of ovarian stimulation individualized and tailored to each woman undergoing IUI [26–28].

Thus, the data concerning the efficacy of ovarian stimulation and its relationship to follicle size as well as the endometrial thickness, are still inconsistent and unclear [20–29]. In addition, there are currently no data correlating the type of ovarian stimulation, follicle size, and endometrial thickness simultaneously to pregnancy rates in fertility clinics in Indonesia, particularly in Bandung, such as the Aster Clinic, Dr. Hasan Sadikin General Hospital Bandung and *Bandung Fertility Center*. This study was performed to observe the relationship between follicle size, endometrial thickness, and types of ovarian stimulation (Clomiphene citrate/CC vs Letrozole) with pregnancy rate especially biochemical pregnancy rate in IUI.

## Materials and methods

### Design of the study and subject recruitment

This was a case–control study designed to examine the association between follicle size, endometrial thickness, and based on the type of ovarian stimulations with Clomiphene citrate (CC) and Letrozole in women undergoing IUI with biochemical pregnancy rate as the primary outcome and correlation between variables as a secondary outcome. Based on local and national guidelines as well as previous studies, Letrozole was given orally in a dose of 2.5 mg, 5 mg, and 7.5 mg, respectively, while CC was given orally in a dose of started of a low dose of 50 mg until a maximum of 150 mg/day. If the patient displayed no response, the dosage was increased [23–28, 30–35].The brain's pituitary gland secretes more follicle stimulating hormone (FSH) and luteinizing hormone (LH) when CC is taken. This action stimulates the growth of the ovarian follicle and thus initiates ovulation. In the other side, Letrozole is a third-generation aromatase inhibitor that works by inhibiting the production of estrogen, causing an increase in the release of gonadotropin-releasing hormone (GnRH) from the pituitary gland, leading to hypoestrogenic condition, negative-feedback mechanism and increased gonadotropin secretion and stimulation of ovarian follicle development. [28, 30–35] The data used were secondary data extracted from the medical records of patients at Aster Clinic, Dr. Hasan Sadikin General Hospital Bandung and *Bandung Fertility Center*, starting from December 2021 until minimum number of samples was fulfilled using a consecutive sampling method.

Women with UI (no abnormalities in male partner’s sperms, anatomical aspect of reproductive system, and hormonal function), aged 20 to 40 years who had undergone IUI programs at Aster Clinic, Dr. Hasan Sadikin General Hospital Bandung and *Bandung Fertility Center* for a maximum of three cycles were included in this study. Incomplete medical record data, poor patient compliance, or complications with treatment as well as dropped-out patients were excluded from this study.

### Ethical aspect and research approval

The data collection at Aster Clinic and *Bandung Fertility Center* was categorized as low-risk as it was conducted using medical record data. After receiving approval and recommendations from the Ethics Committee Review Board of Hasan Sadikin General Hospital—Faculty of Medicine, Universitas Padjadjaran, all procedures were performed in accordance with applicable guidelines and regulations, with reference number LB.02.01/X.6.5.176/2021.

### Data analysis

If normally distributed, the data were analyzed using an independent T-test; otherwise, the Mann–Whitney test would be used. The logistic regression method would be utilized for multivariate analysis. A P-value of < 0.05 was considered statistically significant.

## Results

### Subject characteristics

As shown in Table 1, a total of 122 subjects were analyzed in this study. Subjects aged 20-30 years in the CC group were 21 people (47.7%), and 23 people (52.3%) in the Letrozole group. Subjects aged 31–40 in the CC group were 40 (51.3%) and 38 (48.7%) in the Letrozole group. The difference in patients’ age both in CC and Letrozole group was not statistically significant (P= 0.706). The total mean of body mass index (BMI) was 23.29 ± 3.72 kg/m^2^ with no statistically significant difference between BMI in CC and Letrozole group (P=0.192). There were 17 subjects who had undergone 1 cycle, 54 subjects with 2 cycles, and 51 subjects who had undergone 3 cycles. The difference in number of cycles in each group was considered not statistically significant (P=0.857). Length of marriage in the CC group had an average score of 4.39 ± 1.93, and the Letrozole group had an average score of 4.24 ± 2.39. The difference in the average length of marriage in the CC and Letrozole groups was not statistically significant (P=0.250) It can be concluded that the demographic characteristics between the CC group and the Letrozole group were homogenous.

**Table 1:** Subject Characteristics

Variable	Total (N = 122)	CC (n(%))	Letrozole (n(%))	P value
Age				
20–30 years	44	21 (47.7)	23 (52.3)	0.706
31–40 years	78	40 (51.3)	38 (48.7)	
Body Mass Index (BMI) (kg/m^2^)				
Mean $\pm$ SD	23.29 ± 3.72	23.88 ± 3.70	22.91 ± 3.71	0.192
Median	22.10	24.50	21.30	
Range(Min–Max)	13.92 (18.10–32.02)	13.92 (18.10–32.02)	12.80 (18.30–31.10)	
Number of Cycle				
1	17	9 (14.8)	8 (13.1)	0.857
2	54	28 (45.9)	26 (42.6)	
3	51	24 (39.3)	27 (44.3)	
Length of Marriage (years)	122			
Mean $\pm$ SD	4.32 ± 2.17	4.39 ± 1.93	4.24 ± 2.39	0.250
Median	4.00	4.00	4.00	
Range (Min–Max)	11.0 (2.00–3.00)	9.0 (2.00–11.00)	11.0 (2.00–3.00)	

If normally distributed, the data were compared using independent T-test; otherwise, the Mann–Whitney test would be used. A P-value of < 0.05 was considered statistically significant (CI = 95%)

### The association between follicle size, endometrial thickness, and type of ovarian stimulations to biochemical pregnancy rate outcome

Based on Table 2, follicular size between 18 and 22 mm was associated with a higher biochemical pregnancy rate (76.8%) in 74 individuals rather than follicle size of ≥ 22 mm in this study. There was a statistically significant correlation between follicle size of 18–22 mm and biochemical pregnancy rates (P = 0.001) in both CC and Letrozole groups.

**Table 2:** Relationship between follicle size, endometrial thickness, and type of intervention to pregnancy rate outcome

Variable	Total (N = 122)	Outcome pregnancy rate		OR (CI = 95%)	P *value*
		Pregnant	Not pregnant		
Follicle size (mm)					
18–22 mm	74 (60.7)	43 (76.8)	31 (47)	Ref. 3.734 (1.701–8.199)	0.001*
≥ 22 mm	48 (39.3)	13 (23.2)	35 (53.0)		
Endometrial thickness (mm)					
< 8 mm	76 (62.3)	33 (58.90)	43 (65.2)	Ref.	
8–10 mm	41 (33.6)	20 (35.7)	21 (31.8)	0.512 (0.081–3.240)	0.477
≥ 10 mm	5 (4,1)	3 (5,4)	2 (3,0)	0.635 (0.069–4.270)	0.638
Types of ovarian stimulation					
CC	61	22 (36.1)	39 (63.9)	Ref.	0.030*
Letrozole	61	34 (55.7)	27 (44.3)	2.232 (1.079–4.618)	

If normally distributed, the data were compared using independent T-test; otherwise, the Mann–Whitney test would be used. A P-value of < 0.05 was considered statistically significant (CI = 95%)

* P<0.05 was considered statistically significant

Overall, 62.3% of women had an endometrial thickness of < 8 mm, 33.6% had 8–10 mm endometrial thickness, and only 4.1% women had endometrial thickness of > 10 mm. No statistically significant difference in biochemical pregnancy rates based on endometrial thickness in CC and Letrozole group.

Based on the types of ovarian stimulation, 55.7% in the Letrozole group became pregnant, compared to 36.1% subjects in the CC group (P = 0.03). Therefore, it is possible to conclude that the type of Letrozole intervention is a factor that increases pregnancy rates.

### Multivariate analysis

The follicle size variable and the types of ovarian stimulation had P-value of < 0.25, hence they were included for multivariate analysis (Table 3). As a result, follicle size influenced the pregnancy rate in IUI.

**Table 3:** Multivariate analysis

Variable	B	SE	OR (CI = 95%)	P *value*
Follicle size	1,283	0.407	3.606 (1.623–8.011)	0.002*
Type of ovarian stimulations	0.748	0.388	2.274 (1.082–4.777)	0.054
Constant	− 1.349	0.386		

Multivariate test analysis was performed by logistic regression test (95% CI). P-value of < 0.05 was considered statistically significant

* P<0.05 was considered statistically significant

According to the type of ovarian stimulations, Clomiphene Citrate (CC) produced a follicle size of 18–22 mm by 45.9%. Moreover, Letrozole led to 54.1% follicle size of 18–22 mm (P = 0.026). Letrozole also enhanced the likelihood of biochemical pregnancy rates 1.513 times more than CC (Table 4).

**Table 4:** Relationship between follicle size and type of stimulation and pregnancy rate

Variable	Total (N = 122)	Size Follicle		OR (95%CI)	*P* value
		> 22 mm	18- 22 mm		
Ovarian stimulations					
Clomiphene citrate	61 (50.0)	27(56.3)	34(45.9)	Ref.	0.026*
Letrozole	61 (50.0)	21(43.8)	40(54.1)	1.513(0.728–3.142)	

If normally distributed, the data were compared using independent T-test; otherwise, the Mann–Whitney test would be used. A P-value of < 0.05 was considered statistically significant (CI = 95%)

* P<0.05 was considered statistically significant

## Discussion

### Follicle size

Follicular size reflects follicle maturation and has been shown to be associated with the success rate of IUI. Follicle size that is too small or too large reduces the success rate of IUI. The study by Hancock et al., examined 1676 IUI cycles and found that follicle size 21.1–22 mm was associated with a higher probability of clinical pregnancy. In this study, the size of the dominant follicle was an independent predictor of clinical pregnancy rate. Another study by Shalom-Paz et al., found that the mean follicle size of the conception group was 20.4 1.2 mm compared to the follicle size of the non-conception group of 18.8 $\pm$ 1.9 mm. Palatnik, et al. found that pregnancy rates increased in dominant follicle size by 23–28 mm [19, 28, 29]. The Palatnik et al. study also suggested that optimal dominant follicle size (and an increase in follicle size of 0.5 mm to the optimal point) was associated with an increase in endometrial thickness, resulting in a higher probability of pregnancy rate. This finding is in line with the study conducted by Iberico et al., who found that pre-ovulatory dominant follicle size > 15 mm was associated with better pregnancy rates than follicle size 15 mm [30, 31]. These studies are in line with the results of this study which showed that the optimal follicle size to increase the chances of pregnancy rate ranged between 18 and 22 mm in the two types of ovarian stimulation groups.

### Endometrial thickness

A study by Palatnik et al. stated that pregnancy rates were higher in women who were found to have larger follicle size, which was also followed by thicker endometrial thickness. In women with smaller or thinner follicular size and endometrial thickness, the pregnancy rate was recorded lower. This indicates that there was a correlation between follicular growth and the development of endometrial thickness. Yavuz, et al. stated that an endometrial thickness of 8 mm resulted in a high clinical pregnancy rate, whereas according to Kovac et al. an endometrial thickness of 10 mm is associated with better clinical pregnancy rates [36, 37]. Available data regarding optimal endometrial thickness to support pregnancy rates are inconclusive. This is in line with the results in this study where endometrial thickness was not a significant variable.

### The types of ovarian stimulation

Intrauterine insemination can be carried out with various ovulation stimuli, such as CC and letrozole. Both have different effects and characteristics from each other. Clomiphene citrate has long been the first-line therapy for various ovulatory disorders such as in the case of UI, but CC has often unwanted peripheral anti-estrogenic effects. Resistance to CC can also be a factor in IUI failure. This resistance is found in 15–40% of women [20]. Letrozole is also one of the first-line options for UI therapy. In this study, Letrozole increased the odds of pregnancy by 1.1513 times higher than CC.

Letrozole is a third-generation aromatase inhibitor that works by inhibiting the production of estrogen, causing an increase in the release of gonadotropin-releasing hormone (GnRH) from the pituitary gland [20–22, 35]. Letrozole is highly selective and several studies have shown good pregnancy rates, cost-effectiveness, lower side effects, and better patient compliance. In more detail, letrozole works by inhibiting the aromatase enzyme by competitively binding and causing a decrease in estrogen biosynthesis in all tissues. This hypo-estrogenic condition causes the release of the hypothalamic/pituitary axis from negative feedback mechanisms leading to increased gonadotropin secretion and stimulation of ovarian follicle development. Letrozole itself has recently been studied and found to be more effective than CC [20–28]. This may be explained by the nature of letrozole which has a fast half-life, which is only 48 h (much faster than CC, which is two weeks). The use of letrozole was found to have a better effect on the condition of cervical mucus and endometrial thickness to support sub-endometrial and intra-endometrial vasculature, both of which have a significant effect on embryo implantation and pregnancy rates [6, 20–22]. Davar, et al. found that letrozole produced a biochemical pregnancy rate of 8.3% and CC produced a chemical pregnancy rate of 5.5% [6, 20–22, 32–34]. The results shown from previous studies are in line with the results in this study.

## Conclusion

The follicle size of 18–22 mm and using Letrozole rather than CC as ovarian stimulators are predictive factors associated with a higher pregnancy rate.

## Limitations

Nevertheless, this study also has several limitations, including case–control methods, and has not been carried out in a randomized controlled trial and double-blind methods, so it only relies on medical record data which can still cause bias. The type of intervention in this study was only grouped based on the administration of CC and Letrozole without regard to the dosage which could affect the study outcome.

## Data Availability

The authors declare that the personal data from any patients involved in this study will not be shared based on patients’ confidentialities.

## Abbreviations

BMI: Body mass index
CC: Clomiphene citrate
GnRH: Gonadotropin-releasing hormone
IUI: Intrauterine insemination
IUI/COH: Intrauterine insemination with controlled ovarian hyperstimulation
